# A Starting Point for Fluorescence-Based Single-Molecule Measurements in Biomolecular Research

**DOI:** 10.3390/molecules191015824

**Published:** 2014-09-30

**Authors:** Alexander Gust, Adrian Zander, Andreas Gietl, Phil Holzmeister, Sarah Schulz, Birka Lalkens, Philip Tinnefeld, Dina Grohmann

**Affiliations:** Physikalische und Theoretische Chemie - NanoBioSciences, Technische Universität Braunschweig, Hans-Sommer-Strasse 10, Braunschweig 38106, Germany

**Keywords:** single-molecule fluorescence spectroscopy, fluorescence resonance energy transfer, photophysics, photoprotection, fluorescent dye, dynamic heterogeneity

## Abstract

Single-molecule fluorescence techniques are ideally suited to provide information about the structure-function-dynamics relationship of a biomolecule as static and dynamic heterogeneity can be easily detected. However, what type of single-molecule fluorescence technique is suited for which kind of biological question and what are the obstacles on the way to a successful single-molecule microscopy experiment? In this review, we provide practical insights into fluorescence-based single-molecule experiments aiming for scientists who wish to take their experiments to the single-molecule level. We especially focus on fluorescence resonance energy transfer (FRET) experiments as these are a widely employed tool for the investigation of biomolecular mechanisms. We will guide the reader through the most critical steps that determine the success and quality of diffusion-based confocal and immobilization-based total internal reflection fluorescence microscopy. We discuss the specific chemical and photophysical requirements that make fluorescent dyes suitable for single-molecule fluorescence experiments. Most importantly, we review recently emerged photoprotection systems as well as passivation and immobilization strategies that enable the observation of fluorescently labeled molecules under biocompatible conditions. Moreover, we discuss how the optical single-molecule toolkit has been extended in recent years to capture the physiological complexity of a cell making it even more relevant for biological research.

## 1. Introduction

Almost twenty years ago, Yanagida and colleagues published the first fluorescence-based single-molecule study of a biological sample in aqueous conditions at ambient temperatures [1]. Yanagida monitored the turnover of individual ATP molecules by a single myosin in real-time. However, in order to achieve this goal several challenges had to be overcome. First, in order to monitor single molecules in solution they had to implement a microscopy technique that drastically reduces the background of scattering light originating from the solution, dust and optical elements. Using total internal reflection fluorescence (TIRF) microscopy, a reduction of the background signal by a factor of 2000 could be achieved and this crucial step forward ultimately allowed the detection of individual fluorescently labeled myosin proteins. Secondly, in order to monitor the ATP-turnover of myosin two spectrally different fluorophores had to be attached to myosin and ATP, respectively. Fluorescent dyes serve as reporters that inform about the molecular state of the molecule. However, the reporter itself is already complex in nature and its photophysical behavior has to be understood before a conclusion about the state of the biomolecule it is attached to can be drawn. The fluorescence signal of the reporter is the key for the recognition and identification of the biomolecules and provides evidence that indeed a single molecule is observed. The single photobleaching step of the fluorescent signal is a hallmark of an individual fluorophore. On the other hand, photobleaching is an issue if fluorescence signals are to be monitored over an extended period of time. Yanagida was aware of this problem and added photoprotection reagents to the solution to suppress photo-induced oxygen-mediated photobleaching.

Despite the technical challenges, the big advantage of the interrogation at the single-molecule level is that an averaging of all observed parameters is avoided. Thereby, subpopulations, competing reaction pathways and transient intermediate states, which are frequently obscured in an ensemble of molecules, can be identified. By now, many techniques from the standard biochemical and molecular biology repertoire find their counterpart in the single-molecule fluorescence world. For example, co-immunoprecipitation assays can be directly carried out on the single-molecule level in order to detect protein-protein interactions [2]. A recently developed single-molecule technique from the Moerner laboratory exploits the altered motion of a molecule in response to an external electric field to sense the molecular size and charge and by inference the association state of a molecule, a principle well-known from gel electrophoresis analysis of nucleic acids and proteins [3]. Traditionally, cellular DNAs and RNAs have been detected via short complementary DNA or RNA probes (e.g., fluorescence *in situ* hybridization, FISH). Fast *in situ* hybridization (fastFISH) on the single-molecule level relies on the same principle of specific hybridization between an RNA transcript and a labeled probe and allows the detection of a single nascent transcript in real-time at sub-second time resolution [4]. While the standard biochemical techniques provide a fast “yes/no” answer, the single-molecule adaptation is much richer in information. Here, the stoichiometry of a complex can be directly inferred and even transient interactions can be detected applying minimal invasive approaches (“single-molecule pulldown”, see Section 6.3). The real-time observation of a molecule reveals whether a single molecule changes its conformational state over time (a condition referred to as “dynamic heterogeneity”) and can help to dissect the order of assembly of a complex biological system like the ribosome [5,6]. In combination with fluorescence resonance energy transfer (FRET) absolute distances and changes of these distances can be determined. Ultimately, multiple distances can be measured to provide insights into the architecture of structurally uncharacterized biological complexes [7] or to follow complex protein folding landscapes [8].

However, the hurdles Yanagida encountered still pose critical steps on the way to a successful single-molecule experiment. Here, we provide a hands-on guide to single-molecule experiments tackling the practical issues of optical single-molecule microscopy using biomolecular probes. In addition, we present newly emerged concepts and methods in the field, that now enable single-molecule measurements on hitherto inaccessible biological samples like native eukaryotic protein complexes taking the single-molecule approach to the physiological complexity of cells.

## 2. Planning a Biological Single-Molecule Experiment

Not every biological technique is downwardly scalable to the single-molecule level. There are requirements on the biological system under investigation to be suitable for single-molecule experiments. One of the main limitations is the so called “concentration barrier” [9]. The concentration barrier is a limiting factor especially for the investigation of biological interactions as dissociation constants (*K*_D_) of those interactions are often in the micromolar range [9]. This means in practical terms that only biological complexes with a low dissociation constant (e.g., a *K*_D_ below 50 nM) can be investigated as they still exist as complexes even at picomolar to nanomolar concentrations used for single molecule measurements. On the other hand, even if the complex is extremely stable, it is not feasible to go below a lower concentration limit. Even though this might be desirable for the detection of low-abundant biomarkers it will take the molecule a considerable amount of time to diffuse through the detection volume rendering the detection of ultra-low concentrated samples impractical.

**Figure 1:**
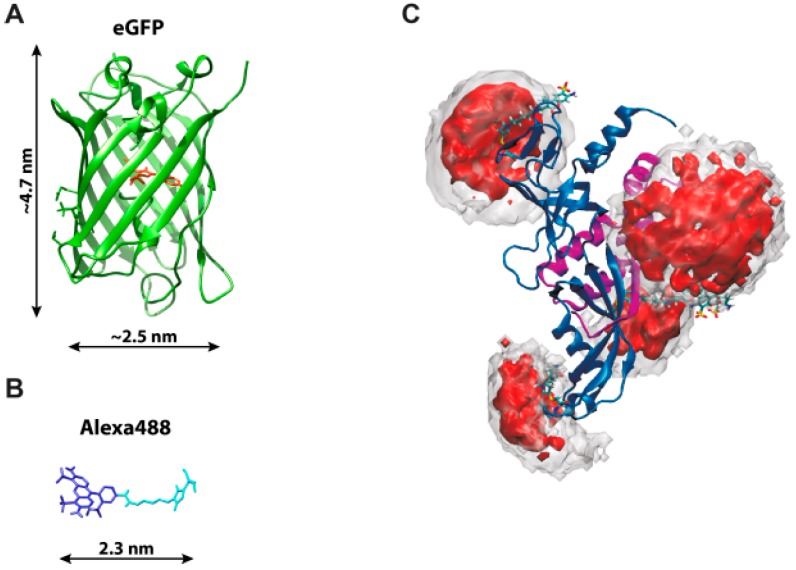
Structure and dimensions of (**A**) the fluorescent protein eGFP (PDB: 4EUL), chromophore highlighted in red); and (**B**) the organic dye Alexa488 (blue) with linker (turquois); (**C**) Coupled to a biomolecule (shown here are RNA polymerase subunits Rpo4/7, PDB: 1GO3, blue and magenta ribbons) the organic dye is restricted in its rotational freedom and samples a defined volume. The dye is attached to different positions of the protein to illustrate how the rotational freedom of the dyes is restricted depending on the protein structure. The conformational space available for the linker-dye molecules can be computed using Monte-Carlo simulations. Clouds envelope 99.5% (gray) and 50% (red) of the total probability (Adapted by permission from PLOS ONE: D. Klose *et al.*, published: 25 June 2012, doi:10.1371/journal.pone.0039492).

Enzymatic reactions can be monitored via a fluorescent substrate that changes its fluorescence properties after turnover. Yet, these kind of fluorescent substrates are available just in rare cases. More often, fluorescence-based single-molecule measurements are employed to follow structural changes in a biomolecule. To this end a modification of biomolecule is require to introduce one or several fluorescent reporters. While fluorescent proteins can be easily attached to any target protein, the poor photophysical performance as well as their large size (Figure 1) renders them unsuitable for most single-molecule applications. Preferably, the advantageous photophysical properties of organic dyes are exploited for single-molecule detection (see Section 3 and Table 1). Site-specific attachment of dyes to proteins requires unique coupling chemistries that exploit for example the thiol group of single cysteines or reactive moieties of unnatural amino acids engineered into the target protein (reviewed for example in [10]). To this end, prior knowledge about the structural organization of the protein is required in order to (i) avoid attachment to structurally or catalytically important residues or amino acids that are involved in protein-protein and/or protein-nucleic acid interaction; (ii) identify surface-exposed amino acids accessible to the reactive dye; (iii) choose a suitable attachment site that does not restrict the rotational freedom of the dye (the orientation factor problem, see Section 3.1) and (iv) place the dyes in an appropriate distance if FRET studies are intended. Efficient and specific labelling demands in most cases the availability of sufficient amounts of pure protein and appropriate purification protocols to efficiently remove excessive dye after the labelling reaction prior to single-molecule measurements. Depending on the question to be addressed, multiple fluorophores have to be incorporated into e.g., different domains of one molecule or different constituents of a large biomolecular complex. This necessitates individually tailored labelling schemes and a prudent dye selection to enable multi-color FRET or co-localization experiments [11,12]. Because of the filtering capabilities of single-molecule approaches, molecules that only carry either the donor or the acceptor can be eliminated from the analysis so that incomplete labeling can be tolerated to a certain extent.

In addition, a downstream activity assay is required to assess whether or a modification of the protein affects functional domains or its catalytic activity.

Advanced single-molecule approaches often require additional information. For example, the single-molecule pull-down (SiMPull) scheme allows the co-purification and single-molecule detection of interaction partners directly from cell extracts [2]. This technique opens up the possibility to visualize complex biological machineries not amenable to *in vitro* assembly. However, it requires prior knowledge about the cellular interaction network in order to fluorescently label relevant proteins [13,14,15,16,17] (see Section 6.3). Another example is the so called “nanopositioning system” developed by Michaelis and coworkers [7,18]. Here, the position of flexible domains or architecture of large macromolecular complexes, which cannot be determined by standard structural biology methods, can be revealed based on multiple quantitative single-pair FRET measurements and triangulation. However, the unknown position cannot be calculated *de novo* but high-resolution structural information of at least one domain of a single protein or one member of the macromolecular complex is required.

**Table 1:** Spectroscopic properties of commonly used organic dyes. Listed are absorption- and emission maximum, fluorescence lifetime and extinction coefficient that characterize the individual dyes ranging from blue to red. Furthermore, the chemical structure of the dye is given and the photophysical behavior is stated.

Fluorophore	Absorption Max (nm)	Emission Max (nm)	Quantum Yield	Fluorescence Lifetime (ns)	Extinction Coefficient (×10^3^ M^−1^·cm^−1^)	Chemical Structure	References	Notes
**Cy2**	489	506	0.12	-	150	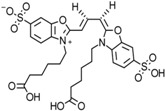	[12,19]	- lower probability of redshifted statecompared toAlexa488, ATTO488
**Dylight488**	493	518	-	-	70	proprietary	-	- phosphine-derivative available for labelling of unnatural amino acids containing an azide moiety
**Alexa 488**	495	519	0.92	4.1	71	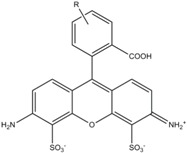	[12,20,21,22,23,24,25,26,27]	- often used to monitor protein folding - donor dye for Alexa 568 and Alexa 594 - red-shifted photobleaching intermediates - quenched by guanosines in close proximity - almost no interaction with DNA
**ATTO 488**	501	523	0.80	4.1	90	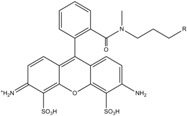	[28,,29]	- redshifted states [11]
**Rhodamine 6G**	525	555	0.95	-	102	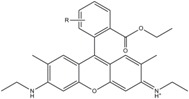	-	-
**Alexa 532**	531	554	0.61	2.5	81	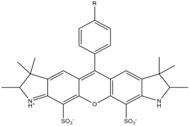	[30]	- spectral fluctuations
**ATTO 532**	532	553	0.90	3.8	115	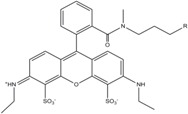	[31]	- high water solubility - good photostability without oxygen removal
**Cy3**	548	562	0.09	0.15	150	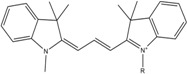	[32,33,34]	- very often used in combination with Cy5 for FRET experiments as the dyes do not interact even if in close proximity to each other - cis-trans-isomerization
**Cy3B**	558	572	0.7	2.8	130	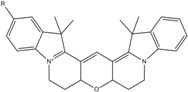	[32,35]	- increased photostability compared to Cy3 due to inhibition of cis-trans-isomerization
**Alexa 546**	556	573	0.79	4.1	104	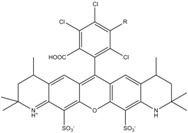	[27]	-
**Alexa 555**	555	565	0.10	0.30	155	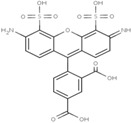	-	-
**DyLight550**	562	576	-	-	-	proprietary	-	- phosphine-derivative available for labelling of unnatural amino acids containing an azide moiety
**ATTO 565**	563	592	0.90	4.0	120	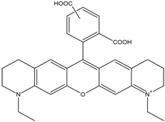	[28]	- used in multi-color FRET schemes
**Alexa 568**	578	603	0.61	3.6	91.3	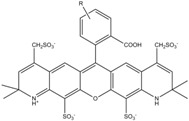	-	-
**Alexa 594**	590	617	0.66	3.9	90	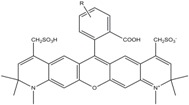	[22,23,24,25,36]	- often used in single-molecule protein folding experiments - acceptor for Alexa488 in FRET experiments
**ATTO 590**	594	624	0.80	3.7	120	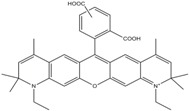	-	-
**Cy5**	646	664	0.27	1.0	250	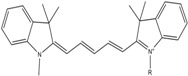	[19,33,34,37,38,39]	- broadening due to spectrally different Cy5 states - switchable with thiols - cis-trans isomerization - ROXS inhibits blinking
**Alexa 647**	650	668	0.33	1.0	239	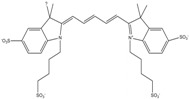	[19,40]	- switchable with thiols—similar to Cy5
**DyLight650**	652	672	-	-	-	proprietary	-	- phosphine-derivative available for labelling of unnatural amino acids containing an azide moiety
**ATTO647N**	644	669	0.65	3.5	150	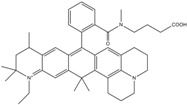	[28,39,41]	- manifold of spectral states - lifetime seems to depend strongly on environment [42,43] - photostability improved by ROXS - heavily quenched if placed close to guanosine bases
**ATTO680**	680	700	0.30	1.7	125	proprietary	[43]	- redshift in absorption and emission maxima with increasing glycerol concentration
**Cy7**	750	773	0.30	0.8	199	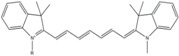	[11,44]	-

## 3. Tuning Photophysical Processes of Dyes for Fluorescence Single-Molecule Detection

**Figure 2:**
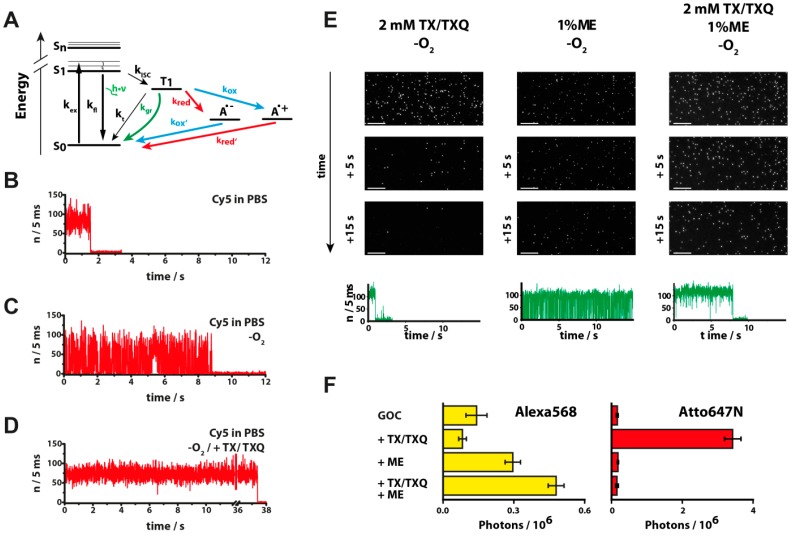
(**A**) Jablonski diagram: once an organic dye absorbs a photon it is excited from the ground state S_0_ to the first excited singlet state S_1_ (k_ex_) and can return to S_0_ by emission of a photon (k_fl_, *fluorescence*). The dye can undergo numerous excitation-emission cycles before it enters a non-fluorescent triplet state T_1_ via intersystem crossing (k_ISC_). Once in the triplet state, the dye can either return directly to S_0_ (k_t_) via different, competing pathways or can be destroyed by singlet oxygen (photobleaching). The reduction of the dye in its triplet state to a non-fluorescent radical anion A^−^ (k_red_, red), which can subsequently be oxidized (k_ox_, blue) promotes the depopulation of T_1_ to S_0_. The depopulation of the triplet state can also start with the oxidation reaction followed by the reduction step; (**B**–**D**) Exemplary photophysical behavior of organic dyes. Fluorescence transients of Cy5-labeled dsDNA in aqueous PBS buffer (B). The removal of oxygen leads to reduced triplet quenching and increased blinking. (C) Addition of 2 mM Trolox(TX)/Troloxquinone (TXQ) enables stable and prolonged fluorescence over minutes (D). Photostabilization by the “geminate recombination” mechanism [48] (**E**). A time series of frames showing the photostability of Alexa568 dyes in different buffers (2 mM TX/TXQ, left column; 1% beta-mercaptoethanol (ME; center), and a combination of both (right column)), oxygen was removed in all cases (see text for details). Scale bar: 10 μm. The last row shows transients from a confocal microscope that illustrate the differences in blinking and photobleaching behavior of the dye. **(F**) Total number of detected photons before photobleaching or long-lived dark states for the dyes Alexa568 and ATTO647N in different buffers (figures in panel A, E and F adapted by permission from Holzmeister *et al.*, Geminate recombination as a photoprotection mechanism for fluorescent dyes, Angewandte Chemie, 2014).

Fluorescence-based single-molecule spectroscopy relies on the detection of fluorescent signals from the sample of interest. The absorption of a photon transfers the fluorophore from an electronic ground state (S_0_) to an excited state (S_1_) (Figure 2A). After relaxing to the lowest vibrational level of S_1_, the molecule can return to the ground state by emitting a photon. The energy of this photon is lower than that of the absorbed photon resulting in a read-shifted emission spectrum, a phenomenon referred to as “Stokes-shift”. Single-molecule fluorescence spectroscopy exploits this fact to spectrally separate the emission light from the excitation light. A further advantage of using fluorescence as quantitative unit of measurement is that different parameters like the spectral properties (absorption and emission of the fluorophore), the fluorescence intensity (“brightness”), the fluorescence lifetime and anisotropy (see Section 3 for details) can be determined providing a wealth of information. These different parameters can be accessed individually or in combination via multi-parameter fluorescence detection [45,46,47]. However, in order to detect the molecule of interest it has to emit a sufficient amount of photons per second upon illumination with light. Even though the fluorescence of intrinsic aromatic residues can be successfully exploited in ensemble fluorescence measurements, these fluorophores are not suitable for fluorescence-based single-molecule studies as they do not emit efficiently at the spectral range generally used in single-molecule spectroscopy.

### 3.1. Structure and Characteristics of Fluorescent Dyes

In the simplest approximation, a fluorescent dye can be described as a small organic molecule characterized by an extended delocalized π-electron system. It can be used as label that robustly reports on the presence and location of a molecule. When attached to a biomolecule, organic dyes can act as a “spy” giving a direct feedback about the molecular status and the behavior of a biomolecule. The fluorophore is characterized by its absorption and emission spectra, by its extinction coefficient ε and by its quantum yield Φ (that is the ratio between emitted fluorescence photons and absorbed photons).

(1) $$\Phi =\frac{\#\;\mathrm{photons emitted}}{\#\;\mathrm{photons absorbed}}$$

**Figure 3:**
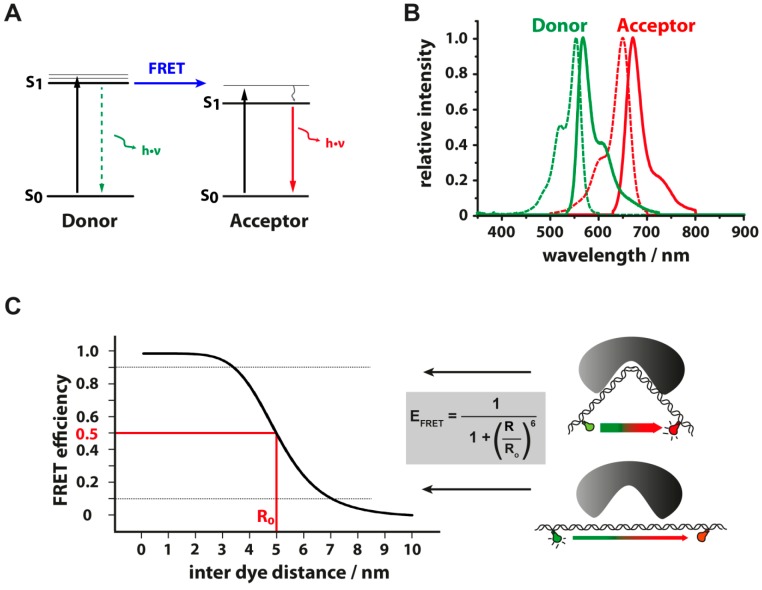
Principles of fluorescence resonance energy transfer (FRET). (**A**) If an excited donor fluorophore is in close proximity (approximately 1–10 nm) to an acceptor fluorophore, radiation-less energy transfer to the acceptor fluorophore via dipole-dipole interaction (FRET) competes with radiative return (photon emission) of the donor to its ground state (dotted arrow). The acceptor, now in its excited state (S_1_), can then return to its ground state (S_0_) by the emission of a photon and the ratio of photons emitted by the donor and acceptor molecule defines the transfer efficiency; (**B**) FRET occurs only if the emission spectrum of the donor dye (green continuous line, Alexa555) overlaps with excitation spectrum of the acceptor dye (red dashed line, Alexa647); (**C**) Distance dependency of the energy transfer efficiency between a donor and acceptor fluorophore (assuming a Förster radius R_0_ of 5 nm). FRET can inform for example about structural changes in the DNA induced by the transcription factor TBP (TATA-binding protein) [49]. Distance changes can be measured most sensitively when working in the dynamic range between approximately 10%–90% FRET efficiency (dotted lines).

The fluorescence brightness is proportional to the product of ε and Φ and is a very important parameter for a fluorophore to be used in single-molecule assays. In FRET assays, the fluorophore serves as donor or acceptor to report on inter-fluorophore distances. Here, the excited donor fluorophore does not relax via photon emission but insteadcouples to the acceptor fluorophore via dipole-dipole interactions. The now excited acceptor fluorophore can return to its ground state by the emission of a photon (Figure 3A). Because the donor and acceptor emit photons at different wavelengths it can be clearly distinguished whether the photon was emitted by the donor or acceptor fluorophore using typical single-molecule detection schemes (see Section 4). As the efficiency of the energy transfer is strongly dependent on the distance between the donor and acceptor fluorophore, FRET can be used to determine inter- and intramolecular distances and changes thereof (see Section 3.3).

A common complication of fluorescent dyes is that they are not ever-glowing light bulbs [50]. Instead, they suffer from photoblinking and photobleaching and often exhibit environmentally sensitive fluorescence (Figure 2). Many other parameters such as water solubility, photostability, redox properties, fluorescence lifetime, and propensity of isomerization, spectral stability, tendency to aggregate, uptake by cells as well as the availability of conjugatable derivatives have to be taken into account. Since these properties are not easily accessed and evaluated by the non-specialist, we have listed some fluorescent dyes that have been successfully used especially in single molecule FRET experiments in Table 1. We provide the structure (if available), spectroscopic properties as well as some remarks and references. While this list is subjective and not exhaustive we would like to provide a guide to facilitate dye selection for certain single-molecule experiments. It is noteworthy that the dye choice strongly depends on the specific application. The environmental sensitivity of fluorescent dyes is not necessarily a disadvantage but can be used for specific sensing applications of e.g., pH [51,52], redox environment [53], proximity of specific electron donors (such as guanosine and tryptophan in biomolecules) [20], and viscosity (recently elegantly used to sense protein proximity [54,55]).

For biological applications, a fluorescent label is ideally a small and water-soluble molecule that avoids aggregation and prevents non-specific interactions with the biomolecule via hydrophobic interactions. Fluorescent dyes used for single-molecule fluorescence applications commonly cover the visual to near infrared spectrum (480–750 nm). They exhibit a maximum extinction coefficient ε_max_ > 80.000 M^−1^·cm^−1^ and a fluorescence quantum yield Φ > 10%. Their fluorescence lifetime is of the order of a few nanoseconds and their size is roughly one to two nanometers.

The relative position of the fluorophore with respect to the biomolecular structure it has been attached to has to be carefully taken into account in order to (i) understand the photophysical behavior of the fluorophore; (ii) determine accurate inter-dye distances via FRET measurements; and (iii) to build and validate structural models. Anchored fluorophores have to be understood as flexible molecules rather than static entities with a fixed position (Figure 1C). The linker to whom the fluorophore is attached can adopt numerous conformations that sample a defined volume, and the mobility strongly depends on the length and flexibility of the fluorophore linker. Furthermore, the accessible volume can be restricted by the surrounding biomolecule. Long linkers (up to 15 Å in length) have been introduced to avoid electrostatic or hydrophobic interactions between the biomolecule and the dye and to support the rotational freedom of the fluorophore. Nevertheless, fluorescence quenching can be observed for the dye ATTO647N and others attached to nucleic acids that contain the nucleobase guanine in close proximity. Upon van der Waals contact between the organic fluorophore and the nucleobase the fluorescence is quenched, which results in a multi-exponential fluorescence decay of fluorescence lifetime and consequently a heavily reduced fluorescence intensity [21]. Guanine serves as electron-donating moiety that allows photo-induced electron transfer (PET) reactions with the dye [20,56]. It is also known that the cyanine dye Cy5 changes its fluorescence properties upon cis-trans isomerization, which is influenced by solvent effects. The fluorescence lifetime of ATTO647N differs depending on the protein environment [38,42,43].

**Figure 4:**
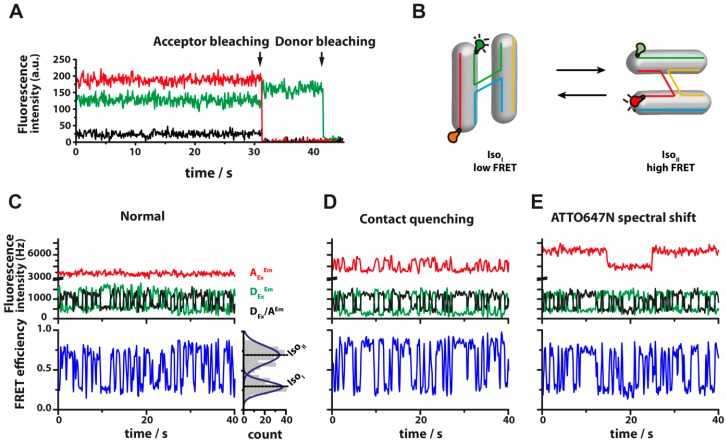
(**A**) Typical fluorescence transients of immobilized dsDNA labeled with a donor (Cy3B) and an acceptor dye (ATTO647N) allowing FRET. Using alternating laser excitation the acceptor emission upon acceptor excitation is recorded in addition to the FRET signal. The donor fluorescence is shown in green, the acceptor in red and the FRET intensity in black, respectively. Upon bleaching of the acceptor dye (32 s) no fluorescence is emitted by the acceptor and the donor fluorescence increases because of the inhibited energy transfer. Simultaneously, the FRET efficiency is reduced to zero. (**B**) The Holliday Junction (HJ) is composed of four single stranded DNAs that can adopt two different conformations, Iso_I_ or Iso_II_ (B). (**C**) Fluorescence time transient of a Cy3B-ATTO647N labeled HJ that undergoes conformational changes (TIRF measurement). The HJ is immobilized on a PEG surface in PBS with 200 mM MgCl_2_ added to the buffer. The FRET efficiency (lower trace, blue) changes rapidly between a low FRET and a high FRET state and at the same time Cy3B and ATTO647N fluorescence intensities show an anti-correlated behavior (upper trace, donor in green, acceptor in red, FRET in blue) until the acceptor bleaches. A double-Gaussian fit of the histogram reveals FRET values E_low FRET_ = 0.29 for conformation Iso_I_ and E_high FRET_ = 0.70 for conformation Iso_II_; (**D**) The fluorescence of ATTO647N is quenched when it can directly interact with the donor fluorophore Cy3B. The quenching effect leads to a slightly decreased FRET efficiency [59] as compared to the FRET efficiency retrieved from a HJ with the FRET pair Cy3/Cy5 (a dye pair known to not directly interact); (**E**) ATTO647N can change its emission characteristics due to spectral shifts [39] potentially causing deviations in the FRET efficiency as shown by Di Fiori *et al.* [59].

Beyond these simple parameters, fluorescent dyes possess more specific characters. ATTO647N, for example, is one of the most prominent fluorescent dyes, probably the brightest and most photostable dye in the near-infrared spectral region [39]. It has been used in some stunning single-molecule and superresolution experiments and an extremely high localization accuracy can be achieved when using ATTO647N under physiologically relevant conditions [40,41]. One might wonder why it is not used for almost all single-molecule experiments in this spectral range. The drawbacks are that ATTO647N is comparatively hydrophobic so that it could, for example, not be used for imaging of DNA origami structures by DNA PAINT [57]. It furthermore shows contact quenching with other fluorescent dyes such as TMR and Cy3B [58,59], and it can adopt different spectral forms (Figure 4D,E). Different spectral forms refers to the fact that individual dye molecules change their absorption and emission spectra between different molecules or even dynamically for the same molecule. Spectral fluctuations h were not only be observed for ATTO647N [39] but also for many other dyes, especially rhodamine derivatives (see e.g., [11,12]). Spectral fluctuations can especially impair the precision of single-molecule FRET experiments but are less of an issue if the focus of interest is kinetic information of conformational transitions.

Organic fluorophores fall into different classes depending on their chemical core structure but generally contain a large delocalized π-electron system. Careful inspection of the photophysical characteristics of these dyes made it possible to correlate fluorescence with chemical structure and to identify weaknesses in the dye molecules. In order to improve the spectroscopic properties of the dyes diverse side chains and double bonds at specific positions have been introduced. For example, the cis-trans isomerization of cyanines has been suppressed by the incorporation of three 6-membered rings in the derivative Cy3B (the cis-state is the non-fluorescent state of the molecule) increasing the quantum yield of the molecule from 15% (Cy3) to 67% (Cy3B). Currently, cyanines (e.g., Alexa647 and Cy3, Cy3B, Cy5), xanthene derivatives (fluorescein), oxazines (e.g., ATTO655), carbopyronine (ATTO647N) and rhodamines (e.g., Alexa488, Alexa594, ATTO565, Rhodamine B) are commonly used in single-molecule measurements. Dyes from the same class can often be replaced by another member of the class. A typical example is Cy5 and Alexa647. Both dyes have an identical chromophore but different substituents. The difference is that Alexa647 possesses more sulfonic acid groups at different positions providing a higher water solubility.

### 3.2. Photoblinking and Photobleaching

After a fluorescent dye molecule has absorbed a photon and resides in an excited state it has an increased tendency to undergo chemical reactions. Often these photoreactions involve reactive oxygen species or rearrangements that lead to a destruction of the delocalized π-electron system, *i.e.*, a sudden cease of fluorescence. The photoreaction is visible as a digital, instantaneous drop of the fluorescence intensity. This behavior is shown in Figure 2 for the fluorescent dye Cy5. A single Cy5 molecule is detected with a count rate of about 15 kHz which abruptly drops to the background level after ~1.6 s. Since ambient oxygen in solution is one of the typical photochemical reaction partners of organic compounds especially through the formation of reactive singlet oxygen, oxygen removal by enzymatic systems has become routine for single-molecule assays [60,61]. A typical fluorescence intensity transient of Cy5 after oxygen removal is shown in Figure 2C. The most commonly used oxygen removal systems are glucose oxidase in combination with catalase (GOC) and protocatechuate-dioxygenase (PCD) [62,63] which uses β-d-glucose and 3,4-protocatechuic acid (PCA) as substrates, respectively. Oxygen removal leads to the formation of carboxylic acids like d-gluconic acid and 3,3-carboxy-cis,cis-muconic acid. These oxidation products lead to a drop in the pH value of the measurement medium. However, many biomolecules and also some fluorescent dyes are affected by a pH change. The pH change can potentially impair enzymatic function and dynamic behavior of the biomolecule [64]. For fluorescent dyes the blinking behavior or the fluorescence lifetime can be affected. Therefore, the system has to be buffered to avoid pH-induced fluctuations. An alternative oxygen removal system is pyranose oxidase in combination with catalase (POC) [65]. Here, d-glucose is oxidized to 2-dehydro-d-glucose, a ketone that does not affect the pH but equally well removes oxygen from aqueous buffer solutions.

Even though oxygen removal can significantly increase the photostability of a fluorophore, it in turn can induce fluorescence intensity fluctuations between a “bright state” (on-state) and a non-fluorescent “dark state” (off-state) (Figure 2C,E). The transient off-states represent triplet states or other intermediate states such as radical ion states. These off-states are quickly quenched in the presence of oxygen (Figure 2A). The combination of a reductant/oxidant (ROXS) pair ensures that several possible transient dark states including triplet states as well as radical anionic and radical cationic states can be depopulated [39,53]. The swift depopulation of transient dark states additionally blocks photobleaching pathways so that the photostability of the dye is further improved. Figure 2D shows a corresponding fluorescence transient after oxygen removal and addition of the dark state quencher combination Trolox/Troloxquinone (TX/TXQ) [33,53]. Overall, the photostability of e.g., Cy5 bound to DNA is improved by some orders of magnitude compared to ambient phosphate buffered saline (PBS) [66].

While such ROXS buffers improve the photostability of a large number of frequently used dyes (e.g., Alexa647, ATTO647N or the popular FRET pair Cy3/Cy5), their performance is less satisfactory for others, mostly those excited below 600 nm. In some of these cases, like shown for Alexa568 (Figure 2), photoprotection can be improved by a geminate recombination (GR) mechanism [48]. Here, the photostability is increased by reduction combined with an efficient back electron transfer by the same molecule, which is trapped within the solvent cage of the fluorophore. This mechanism requires only a single thiol compound like β-mercaptoethanol (ME) and reduces the triplet lifetime while potentially reactive intermediate states (radical ion states) are largely avoided (Figure 2A). After enzymatic removal of oxygen and in presence of an oxidizing/reducing system like TX/TXQ the dye Alexa568 shows stable fluorescence emission without blinking but bleaches quickly (Figure 2E). In presence of ME instead of TX/TXQ the dye turns more photostable but shows frequent blinking. As ME is a reductant, it is believed that the dark states are based on radical anions formed by photoinduced reduction of the triplet state because the efficiency of geminate recombination is not 100% [48,52]. The GR-ROXS system (a combination of both, ME and TX/TXQ) leads to the desired high photostability and concurrent reduced blinking of dyes like Alexa488, ATTO532, Alexa 532 and Alexa 568 (Figure 2F). Since thiols additionally cause the formation of long-lived dark states that are exploited in superresolution microscopy [67,68] the benefit of adding them for photostabilizing differs from dye to dye (Figure 2F).

### 3.3. Energy Transfer between Fluorescent Dyes

Fluorescence resonance energy transfer (FRET) is one of the most popular fluorescence-based methods in biology as it allows the determination of inter-fluorophore distances and changes thereof and consequently has been widely referred to as “spectroscopic ruler” (Figure 3 and Figure 4). The energy transfer efficiency is dependent on the power of minus 6 of the distance between donor and acceptor fluorophore and is defined as:

(2) $$E=\frac{1}{1+{\left(\frac{R}{R_{0}}\right)}^{6}}$$

R stands for the inter dye distance and R_0_ denotes the Förster radius, at which E = 0.5. R_0_ is defined as:

(3) $$R_{0}=9780{\left(n^{-4}\cdot \kappa^{2}\cdot \Phi_{D}\cdot J\right)}^{\frac{1}{6}}\text{Å}$$

Here *n* denotes the refractive index of the buffer (typically 1.33 for aquoeos solutions), κ^2^ is the orientation factor of fluorophores’ dipoles, Φ_D_ is the quantum yield of the donor in the absence of the acceptor and *J* the overlap integral between the donor emission and acceptor excitation spectrum. The most critical factor for the Förster radius is expressed in the orientation factor κ^2^, which defines the relative orientation of the fluorophores to each other. FRET finds its maximum when the dipole-dipole transition moments of the chromophores are aligned head to tail (κ^2^ = 4) and minimized if they are orthogonal (κ^2^ = 0). When donor and acceptor are freely diffusing and rotating in aqueous solution at room temperature κ^2^ can be approximated to 2/3. The accurate calculation of the orientation of chromophore dipoles to each other and therefore the exact distance between the fluorophores is a challenging task. Errors of 25%, 50%, and 100% in κ^2^ correspond to errors of 5%, 10%, and 15%, respectively, in R_0_ [69] whereas the estimated combined contribution of random errors in non- κ^2^ terms of R_0_ is 10% [70].

Long linkers permit an isotropic distribution of the fluorophore orientation (thereby reducing the κ^2^ problem) but can account for up to 20 Å difference in the inter-dye distance [71]. It is important to keep in mind that the actual distance derived from FRET measurements is the distance between the center of the fluorophores. For this reason, a reliable determination of short distances (<25 Å) via FRET using organic dyes with long linkers cannot be carried out. In contrast, short linkers lead to defined dye positions with a small contribution of the linker to the measured distance but at the cost of reduced rotational freedom, which renders the calculation of κ^2^ more difficult. In first approximation anisotropy measurements can inform about whether or not the fluorophores are freely rotating molecules [72] (fluorescence anisotropy of less than 0.2 is normally assigned to a κ^2^ value of 2/3). The motion of the fluorophore can be severely restricted due to interactions with the biomolecule or energetically favored fluorophore structures. Moreover, an altered photophysical behavior of organic dyes has been reported if the inter-dye distance is too small. At short donor-acceptor separations (e.g., less than 8 bp on DNA) dye-dye interactions have been reported for TMR-ATTO647N, Cy3-ATTO647N and TMR-Cy5 that lead to a quenching of both dyes and an apparent reduction of the average FRET [59]. The donor-acceptor combination of Cy3-Cy5 attached to DNA did not show this behavior making it suitable for studies that require a FRET pair at short distances or where the labels are allowed to collide. Quenching effects have to be considered e.g., in FRET experiments as the quantum yield of the donor contributes to the Förster radius (Equation (3)). In order to avoid erroneous inter-dye distance calculations the donor quantum yield has to be determined in the presence of the individual biomolecule to retrieve the correct R_0_. Even though quenching reduces the brightness of fluorescent dyes and introduces additional uncertainties for FRET-based distance calculations it can also be exploited to monitor conformational dynamics in oligonucleotides [20]. In a similar fashion fluorescent organic dyes can report on molecular interactions if the reaction induces a change in the local environment of the dye. In recent years simulation techniques like Monte Carlo (MC) simulations and molecular dynamics (MD) calculations have been employed to enable a reliable positional modeling of the fluorophore and to predict the theoretical inter-fluorophore distance [21,69,71,73]. These calculations are computationally demanding and as a consequence are not a standard procedure so far. If the local structure in proximity of the dye molecule is known the position distribution of the fluorophore can also be quickly calculated. Computational less challenging geometric accessible volume calculations lead to comparable results as the more laborious MD calculations as shown by Sindbert *et al.* [21]. Furthermore, this work demonstrates that short and flexible linkers can be used in cases where the local structure is unknown and the distance between donor and acceptor is very small. Here the κ^2^-related uncertainties are outweighted by the better defined fluorophore position.

On a bigger scale the measurement of multiple FRET-based distances enables modeling of previously structurally uncharacterized dynamic complexes [7,18,47,69,74,75,76,77,78,79,80,81,82,83]. Using triangulation and the distance information gained from FRET experiments the most likely position of a fluorophore with respect to a structurally known part of the complex can be calculated. This information can be used as constraints in molecular docking approaches to model protein or nucleic acid structures. Michaelis and co-workers have developed a Bayesian framework that accounts for uncertainties about the Förster radius, errors in the measured FRET efficiency and uncertainties in the fluorophore position due to the linker flexibility and the excluded volumes [7,18]. The FRET-based structural approach has been extended recently to analyze FRET networks taking into account fluorescence anisotropy data in order to improve localization accuracy [18,74].

## 4. Setups for Fluorescence Based Single-Molecule Measurements

**Figure 5:**
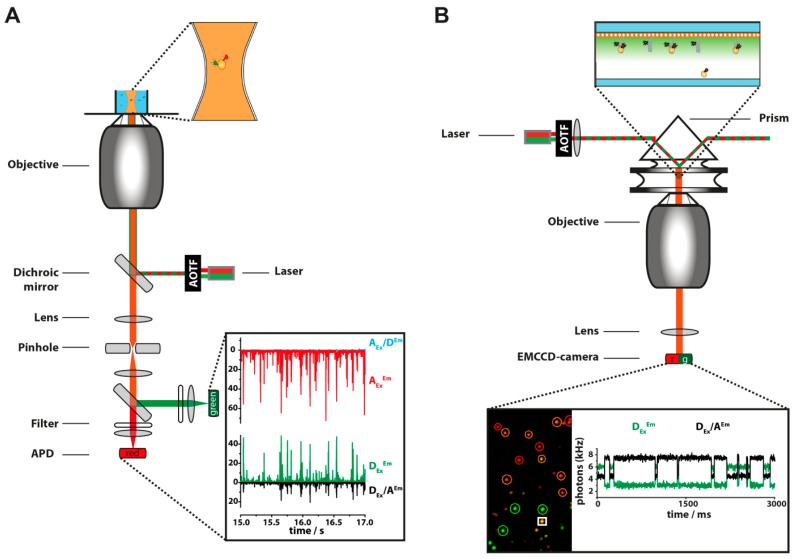
(**A**) Two-color confocal microscopy setup: Green and red excitation lasers are combined and alternated with an acousto-optical tunable filter (AOTF) in order to excite a molecule several times with each laser during its diffusion through the focal volume. The objective focuses the light on a diffraction limited spot in solution. Only molecules that diffuse through this confocal volume are excited. In order to further confine the detection volume, the fluorescence is focused through a pinhole followed by a separation by a dichroic beam splitter onto the donor (green) and acceptor (red) detection channel by appropriate spectral filters. These photons, recorded by the avalanche photodiodes (APDs), can be assigned to four channels: acceptor excitation/donor emission (blue), acceptor excitation/acceptor emission (red), donor excitation/donor emission (green) and donor excitation/acceptor emission (FRET channel, black); (**B**) Two-color total internal fluorescence reflection microscopy. In contrast to confocal microscopy an area (approximately 50 × 50 µm^2^) is illuminated which requires higher laser power. After combining and alternating the lasers, their beams are focused by a lens and refracted by a prism (incident angle has to be larger than 63°) onto the upper side of a flow chamber. This way an exponentially decaying evanescent field is generated and only molecules in close proximity to the quartz slide are excited. The resulting fluorescence is collected by a water objective and directed to a beam splitter, which separates the fluorescence according to their wavelength. A lens-mirror system projects the donor and the acceptor fluorescence spatially shifted onto an EMCCD camera chip. By software aided superposition of both areas, subpopulations can be identified (green circles: donor only, red circles: acceptor only, orange circles: donor and acceptor) and the fluorescence signal of the molecules can be monitored over time (signal of the molecule in the white square).

Two different approaches are commonly used to reach the required signal-to-noise ratio (SNR) for single-molecule detection: confocal fluorescence microscopy [84,85] and total internal reflection fluorescence (TIRF) microscopy [86,87] (for an overview of the advantages and applications of the respective technique see Table 2). These methods achieve the required SNR by confining light in different ways. In confocal microscopy a laser beam is focused by a high NA microscope objective to a diffraction-limited volume in the same plane (Figure 5A). The diameter of the focus is described for example by the Sparrow criterion [88] (d_s_ = 0.51λ/NA) correlating to a femtoliter excitation volume. In confocal microscopy the excitation and emission light is collected through the same objective (“epi-fluorescence”). A micrometer-sized pinhole positioned in the emission light pathway creates furthermore a diffraction-limited detection volume. Because of the small excited and detected volume the background signal, which scales with the illuminated volume, is reduced. A high-NA objective enhances the SNR further as it allows the collection of a greater number of photons. In order to ensure, that only one molecule occupies the confocal spot, concentrations in the picomolar range are used. The transit of a fluorescently-labeled molecule diffusing through the confocal volume can be detected as a photon “burst”. While a diffusing molecule only spends about 1 ms in the confocal volume a few dozens to a few thousand photons can be detected from an efficient emitter. This provides enough photons to identify the molecule and to extract for example information about stoichiometry and/or FRET efficiency [89]. Data can also be collected from immobilized molecules if a scanning stage is integrated. In both cases the emitted photons are commonly detected using avalanche photo diodes (APDs) that are characterized by a high collection efficiency (on average 70% in APDs compared to 15%–25% in photo multiplier tubes), fast response time, and high gain at low dark count. Typically, the time resolution of APDs is in the range of some 10 to 100 picoseconds. For FRET measurements the emission signal collected from the observation volume is separated into donor and acceptor channels. This is achieved by a dichroic mirror, optical filters and separate detectors to monitor the photon count for the two channels simultaneously. In an advanced excitation scheme called “alternating laser excitation” (ALEX) [90,91] or “pulsed interleaved excitation” (PIE) [92] the donor and acceptor excitation laser alternate in order to separately excite the donor and acceptor molecule. Although developed first for confocal measurements, ALEX can also be implemented in TIRF microscopy. For ALEX modulatable lasers or an accousto-optical tunable filter (AOTF) are part of the setup to produce well-defined alternation of donor and acceptor excitation. Using ALEX, four different fluorescence emission signals can be recorded from a single molecule: the donor emission signal upon donor excitation (D_Ex_^Em^) or upon acceptor excitation (A_Ex_/D^Em^) and acceptor emission upon donor excitation (D_Ex_/A^Em^, “FRET signal”) or upon acceptor excitation (A_Ex_^Em^) (see Figure 4 and Figure 5). This data set not only allows the calculation of the FRET efficiency but can be used to verify the presence of both, the donor and acceptor fluorophore, in the observed molecule (for details see Section 6). It moreover allows the fluorescence-aided sorting of the molecules according to defined search parameters, e.g., (i) the presence of donor and acceptor (ii) fluorescence stability of the fluorophores and (iii) the FRET efficiency of the detected molecule.

**Table 2:** Fluorescence-based methods commonly used to analyze biomolecular interactions, stoichiometries and dynamics in the nanosecond to minute time range. Commonly, confocal fluorescence spectroscopy and total internal reflection (TIRF) spectroscopy are used to analyze the dynamics of single molecules and to measure intra- or intermolecular distances via FRET.

Fluorescence-Based Method		Time Scale	Advantages	Disadvantages
**Confocal fluorescence spectroscopy**	in solution	ns–ms	- molecule sorting for donor- and/or acceptor-labeled species via alternating laser excitation (ALEX) possible - measurements on freely diffusing and immobilized molecules possible	- limited concentration range of labeled species (5–200 pM) - time resolution is limited by the diffusion-time in the focus
	immobilized	ns–min	- fluorescence lifetimes measurements possible - kinetic information	- interactions with the surface due to immobilization might negatively influence activity of the biomolecule - sequential measurements
**Total internal reflection fluorescence microscopy (TIRF)**		ms–min	- molecule sorting for donor- and/or acceptor-labeled species via ALEX possible - parallel measurement of hundreds of individual molecules - spatial information on the observed molecules - able to monitor not synchronized or rare events	- interactions with the surface due to immobilization might negatively influence activity of the biomolecule - no fluorescence lifetime measurements possible

In contrast, the confinement of the illuminated volume in TIRF microscopy is achieved by an evanescent wave at the interface of the cover slip and sample brought about by the total internal reflection of the laser beam. TIRF can be generated by either coupling the laser beam through the extreme edge of a high-NA objective (“objective TIRF”) or by coupling it into a prism above the microscope coverslip (“prism TIRF”) to achieve the angle for total internal reflection (Figure 5B). Here, the refractive index of the sample must be less than that of the cover slip in order to allow total TIRF. The evanescent wave penetrates into the sample and the intensity of the excitation field follows an exponential decay, which extends only approximately 100–200 nm into the low-index medium. Only fluorophores situated in the evanescent field are excited. Hence, the background signal is dramatically reduced in this geometry. TIRF microscopy belongs to the so called wide-field methods. For these methods an area of several 10 microns in diameter is illuminated and the full field of a view is captured frame by frame using electron multiplying charge-coupled device (EMCCD) cameras. TypicallyEMCCD cameras are characterized by a high quantum efficiency (QE > 95% within the visible range) at relatively low dark-count rates. The main advantages of wide-field measurements are that multiple individual spatially separated fluorophores can be observed in parallel and in real-time and not only a single molecule. However, the time resolution that can be achieved in TIRF microscopy does not exceed below the millisecond range when recording with an EMCCD. The limiting factor is the maximum frame rate for EMCCD cameras which are slower than the response time for single photon counting detectors (SPCD, e.g., single photon avalanche photodiodes, SPAD with a QE > 65% in the visible range) commonly used for confocal microscopy. New scientific complementary metal oxide semiconductor (sCMOS) based cameras are cheaper, offer higher frame-rates (*ca.* 100 Hz) at higher image resolution but still suffer from a comparably low quantum efficiency of about 55% [93]. Advances in the semiconductor field recently enabled manufacturer to develop small two dimensional camera-like arrays with SPC ability. However, the QE of about 40% across the visual spectrum using GaAsP as photocathode is still significantly low [94].

TIRF microscopy allows the long term observation of single molecules to observe dynamic processes. However, this requires the immobilization of the molecule. Photophysical effects like photobleaching or blinking of the organic dye limits the observation time usually not extending beyond the minute range (see Section 3 and Figure 4). For some applications it is possible to enhance the observation time to several tens of minutes by drastically reducing the laser power [95]. Very fast reactions that occur within nanoseconds (e.g., conformational changes or protein folding) are not easily accessible with TIRF microscope.

## 5. Surface Preparation and Passivation for Measurement on Immobilized Molecules

In order to observe single molecules using TIRF microscopy or confocal scanning microscopy the molecules need to be immobilized on a glass slide. The preparation of a surface that allows the specific immobilization of molecules is one of the most critical and time consuming steps in single-molecule spectroscopy experiments. Suitable surface preparation methods include a rigorous glass slide washing and a surface passivation protocol. Impurities on the surface can be strongly fluorescent and this is most pronounced if illuminating in the blue to green spectral range. The result is a high non-specific background fluorescence which is especially an issue if fluorescent labels with a weak fluorescent signal like fluorescent proteins are used. Hence, a common strategy relies on bleaching the molecules that cause the background fluorescence with higher excitation power (approximately 10 kW/cm^2^) before immobilizing the sample. In addition transients that show anti-correlated behavior between donor and acceptor intensity upon donor excitation give ultimate proof that these molecules perform FRET (see Figure 4A for an example). Subsequent to the cleaning the surface is coated with a protective layer to prevent the unspecific adhesion of biomolecules. Many organic fluorophores are hydrophobic in nature and easily attach to unprotected surfaces. Attachment to the naked glass surfaces often leads to the undesired deactivation of the biomolecule and therefore, the passivation layer also introduces a favorable biocompatible environment for the biomolecules. A specific immobilization of the molecules is commonly ensured by biotin, which is embedded in the coating layer. After incubation of neutravidin or streptavidin (with its four biotin binding pockets) before the actual measurement, biotinylated biomolecules can be easily attached to the surface via a biotin-neutravidin-biotin sandwich linkage. The extremely strong and long-lived biotin-neutravidin interaction also ensures that the immobilized molecule does not detach prematurely and therefore can be monitored for minutes to hours.

**Figure 6:**
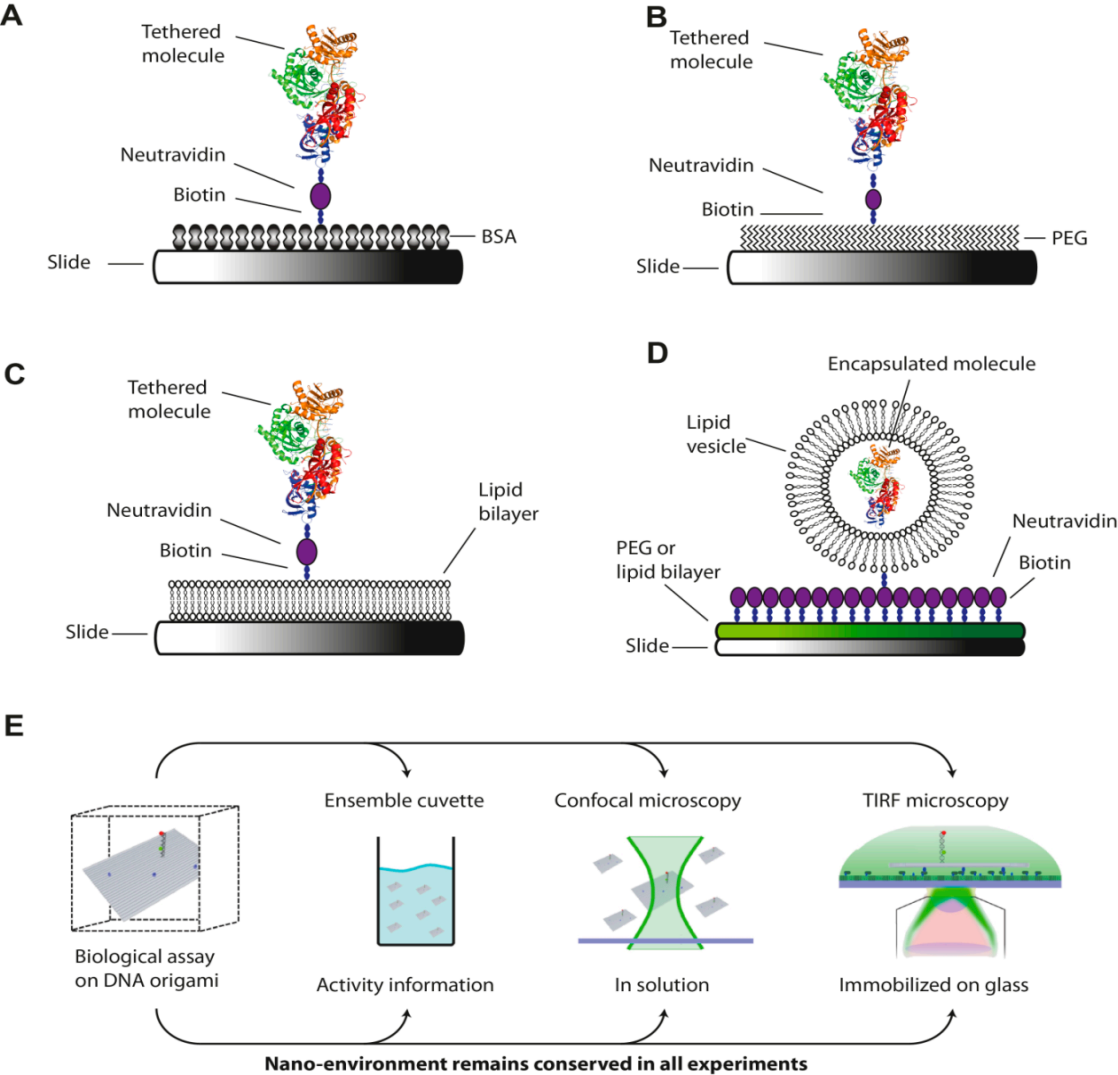
Immobilization and passivation strategies for single-molecule measurements on surface-tethered molecules. Passivation of the glass slide prevents the unspecific attachment of the molecule of interest and is usually realized using biocompatible reagents like (**A**) bovine serum albumin (BSA); (**B**) polyethylene glycol (PEG) or (**C**,**D**) lipids. Mixing of the passivation reagent with its biotinylated counterpart and addition of neutravidin allows the immobilization of the molecule of interest via a biotin-neutravidin linkage on the passivated surface; (**E**) More elaborated strategies make use of pseudo-surfaces like DNA origami. The DNA origami serves as transportable and biocompatible platform that allows the matching of ensemble and single-molecule measurements (see text for details) [104]. (Figure in panel E adapted by permission from Oxford University Press. Gietl, A., *et al.*, 2012 “DNA origami as biocompatible surface to match single-molecule and ensemble experiments”. Nucleic Acids Research 40(14): e110. Figure 1B).

There are different strategies to generate a surface suitable for single-molecule measurements. The most popular approach is blocking the unspecific adsorption of biomolecules to the glass surface using BSA as a pre-adsorbent (Figure 6A). BSA can be used as a blocking solution for hydrophobic and hydrophilic surfaces [96]. Furthermore, in its biotinylated form BSA (BSA/BSA-biotin = 100/1) can serve as attachment point for neutravidin. Often BSA is also added to the reaction buffer to ensure that the surface is still blocked for the duration of the experiment. However the blocking with BSA is not useful in every situation [82]. In many cases, much more complex surface modifications are required to avoid unspecific binding. The most commonly used method to generate an inert surface for assays involving complex biomolecules is the passivation of the glass surface with a self-assembled monolayer (SAM) of polyethylene glycol (PEG). To generate a surface of densely bound PEG molecules (“PEG-brush”) the glass is first silanized (e.g., 2-aminopropyltriethoxysilane) to connect primary amines to the glass surface followed by an incubation with an amino-reactive PEG variant (e.g., *N*-hydroxysuccinimidyl-PEG-Methoxy) [82,97]. Alternatively, silane-PEG-methoxy/Biotin is directly incubated [98]. Mixing methoxy PEG (MW = 3000 to 5000 Dalton) with a defined ratio of biotinylated PEG of approximately 1000/1 allows the placement of neutravidin anchor points to the surface (Figure 6B). To achieve an even better protection against unspecific adsorption BSA is often additionally added to the reaction buffers. To maximize the PEG density and therefore the protection against unspecific adsorption, the passivation with a six-arm, star-shaped PEG molecule has been developed. Due to its shape it can form intermolecular crosslinks and therefore introduces an extremely dense and protective coating against unspecific adsorption [99]. For some applications coating with a PEG-BSA nanogel, a multi-arm PEG cross-linked to BSA, is recommended [100]. It has been shown that the nanogel approach can be a superior protocol for some applications [101,102]. Standard surface passivation protocols have been established for example by the group of Taekjip Ha (a very good step by step protocol to produce clean and well passivated surfaces can be found at [61]. Chandradoss *et al.*, introduced the “double treating” of the surface with PEG and describes critical steps in detail to achieve a high quality surface passivation [103].

Lipid bilayers are an alternative approach to generate a surface which mimics the natural environment of biomolecules in living cells [105,106]. Bilayers can be modified according to the specific needs of the experimenter. Functionalizing of the lipid head with biotin or PEG renders the bilayer compatible for proteins and/or nucleic acids (Figure 6C). The advantage of the lipid bilayer is its uncomplicated handling. In order to generate the bilayer, dehydrated phospholipids are suspended using an aqueous buffer followed by sonication or extrusion through a porous membrane which creates self-assembled vesicles. For the coating the vesicles are injected into a silica flow chamber. Spontaneous fusion leads to the formation of vesicles of a critical size. Once these vesicles rupture a lipid bilayer is formed that coats the entire surface. If solid attachment points are needed, the flow chamber can be also treated with neutravidin first. Neutravidin is unspecifically adsorbed by the surface and forms a very sparse coating. After the coating with a lipid bilayer there will be some isolated molecules of neutravidin distributed among the inert bilayer [106]. However, vesicles can be also used in a whole different manner. It is possible to encapsulate the molecules of interest within a lipid vesicle, which eventually can be tethered to the surface via biotinylated lipids (Figure 6D) [107,108,109]. The difficulty of this technique is to enclose only one molecule of interest into the vesicles. This approach is one way to overcome the concentration barrier of optical single-molecule detection as the effective observation volume is strongly reduced in a vesicle. Typically, vesicles 100 nm in size are used for single-molecule studies, which corresponds to a volume of approximately 1/2000 femtoliter. Hence, a single molecule trapped in the vesicle would have an effective concentration of about 4 µM, a concentration well above the roughly picomolar to the lower nanomolar concentrations normally used for single-molecule spectroscopy.

The direct biotin-neutravidin-anchoring of the molecule is still the standard-procedure for the real-time observation of a molecule but always poses the risk that the functionality of the biomolecule is negatively influenced by the surface. The adverse effects of immobilization have been demonstrated for the refolding of RNaseH and the dynamics of a peptide [110,111]. In both cases the molecules showed a different behavior in ensemble measurements compared to immobilized molecules demonstrating the general immobilization uncertainty. Ideally, the molecule of interest would find an identical environment on the single-molecule surface as it does in ensemble measurements. This idea has been implemented using DNA origami as additional biocompatible adapter surface [104,112]. DNA origami structures are based on a “scaffold” DNA strand (e.g. the single-stranded DNA genome of bacteriophage M13), which can be folded into pre-defined 2D and 3D assemblies at the nanometer scale with the help of hundreds of short oligonucleotides called “staple strands” [113]. Most importantly, DNA origami allows the precise and accurate arrangement of functional molecules like proteins [114], nanoparticles [115], DNA “docking stations” for biomolecular assays [104,116] and fluorescent probes [28]. Given that a functional assay is available, the assay can be set up on the surface of the DNA origami and the functionality of the biomolecule under these conditions can be tested still on the ensemble level. A subsequent transfer to the single-molecule level is easily achieved by simple dilution and immobilization of the DNA origami on the single-molecule surface. The DNA origami acts as transporter and at the same time shields the functional assay from the actual surface used for single-molecule measurements (Figure 6E). Thus, the DNA origami provides not simply a biocompatible surface but can be seen as transfer platform that offers an identical nanoenvironment in ensemble as in single-molecule measurement.

Two alternative approaches that avoid the direct immobilization but allows the long-term observation of a biomolecule were presented recently. The method developed by Tyagi *et al.* is based on a microfluidic platform that can be collapsed to form nanochannels [117]. Here, the molecule is confined to a thin observation field and can be monitored via particle tracking up to 20 s using TIRF microscopy without the need to tether the molecule to the surface. As this method can be parallelized and it provides the additional benefit that it can be operated under oxygen-free conditions in a nitrogen atmosphere, it presents an alternative method to measure FRET-based dynamics whenever it is not possible to immobilize the molecule. Shon *et al.*, introduced a system where molecules are encapsulated in so-called “dimples” also providing a scheme in which single-molecule dynamics can be measured in a parallel fashion without the need for immobilization [118]. In this case, a silica layer that contained an array of nano-fabricated circular depression (“dimples”) was fused to the coverslip. After flushing of the array with the fluorescent sample, the depressions were reversibly sealed with a lid creating a confined femto- to attoliter volume (depending on the manufactured dimple size) corresponding to concentrations between 6.3 nM to 2.2 µM in case a single molecule resides in the confined volume. Traditionally, membrane proteins are difficult to study in fluorescence-based single-molecule assays as they need to be embedded into a lipid bilayer. Watanabe *et al.*, recently reported an elegant method that allows the single-molecule analysis of alpha-hemolysin and the F_0_F_1_-ATP synthase in femotliter chamber arrays [119]. The femtoliter chambers were sealed with a 4 µm diameter lipid bilayer membrane containing individual membrane proteins. The activity of for example a single ATP synthase was monitored using a fluorescent pH sensor monitoring thousands of molecules in parallel using this arrayed lipid bilayer chamber system (ALBiC).

## 6. Data Analysis and Interpretation

### 6.1. Data Registration and Analysis Using Confocal Fluorescence Microscopy

When a molecule labeled with a fluorescent dye transits through the illuminated volume of a confocal microscope the bound dye is excited by the laser beam. The molecule occupies the volume only for a very short time (e.g., in the order of few milliseconds). However, as the excitation and emission in fluorescence occurs within nanoseconds the molecule will be excited multiple times during its passage through the confocal volume. The emission of the dye is recorded photon by photon (assigning each photon a timestamp) by one or more detectors. The time period in which the molecule is detectable depends on different factors like the solvent viscosity, molecule size, path through the volume, and the size of the volume. This compacted burst of photons (see Figure 5A) in a short period of time is used to distinguish the fluorescent signal from the background signal. Different burst search algorithms have been proposed based on for example time-trace binning [84] or interphoton time averaging and thresholding [120]. A commonly used algorithm has been established by Nir *et al.* [89]. Here, a burst is identified if at least *L* successive photons have at least *M* neighboring photons within a time window of length *T*. Typical parameters are *L* = 100, *M* = 30 and *T* = 500 µs. For FRET analysis, the burst search is performed on photons collected from the donor and acceptor channel. The photon burst search takes into account the laser excitation periods if ALEX was used as excitation scheme. This search algorithm is termed the “all-photon-burst-search” (APBS) method [89]. By measuring for several minutes statistics can be built up and information about the heterogeneity of the sample can be gained.

### 6.2. Data Registration and Analysis Using TIRF-Based Single-Molecule Measurements on Immobilized Molecules

In TIRF microscopy a video of the measurement area is acquired using a EM CCD camera. The intensities of spectrally different dyes can be monitored if an image splitter is integrated. The different regions on an EMCCD camera are divided to allow the simultaneous acquisition of emission from two to four spectrally distinct fluorophores. Molecules that diffuse according to Brownian motion only spend <50 ms in the TIR excitation layer [16] and can therefore be easily separated from immobilized molecules which appear as clear diffraction limited spots. However, a certain number of fluorescing spots are always observed, which seems to stem from contaminants from the buffer solution, auto-fluorescing molecules when using cell extracts or impurities in the passivation layer. The opportunity to image spectrally distinct molecules opens up the possibility to detect for example protein-protein interactions simply identified by the co-localization of their emission signals. Recording of the dye bleaching steps even allows to retrieve information about the stoichiometry or oligomerization state of a complex [121]. This elegant tool has been used for example to dissect the stoichiometry of the regulatory splicing complex [122], the stoichiometry at the bacteriophage T7 replication machinery [123] or to reveal the stoichiometry and turnover of the membrane protein MotB that is part of the bacterial flagellar motor [124]. For each dye an individual bleaching step is detectable and the photon count over time curve shows a graph in the shape of a stair (Figure 4A).

TIRF microscopy is a powerful tool to detect and analyze static and dynamic heterogeneity. Figure 6A shows typical fluorescence transients for a biomolecule that carries a donor and acceptor fluorophore. The FRET signal vanishes as soon as the acceptor dye bleaches (32 s) and the donor fluorescence increases as FRET cannot occur any longer. A repetitive process like the fluctuation of a DNA Holliday junction between its two isoforms (Figure 4B–E) are characterized by a rapid switching between two FRET efficiencies while the donor and acceptor fluorescence shows an anti-correlated behavior. Analysis of the time traces provide the full kinetic picture of an interaction and yield information about the lifetime of the complex, the associated dissociation and association rates and finally the dissociation constant [49,125,126,127]. For studies of static heterogeneity, a spatial resolution of two basepairs has been demonstrated using TIRF [128]. The fluorescence intensity traces have to be carefully inspected to make sure that the fluorescence intensities of the donor and acceptor dye are anti-correlated in case dynamic changes are recorded in the FRET channel.

It has to be noted that changes in intensity from a single emitter can be due to changes in many parameters like the aforementioned contact quenching, spectral shifts but also a possible reorientation of the fluorophore if the molecule adopts a new conformation that might lead to polarization effects (e.g., the orientation of the dipoles of the donor and acceptor dye might change dramatically) or a change in quantum yield. Indeed, the photophysics of the acceptor have been identified as major limiting factor for FRET resolution in TIRF microscopy [128]. An example is shown in Figure 4C,D where contact quenching and the spectral shift of ATTO647N attached to a Holliday junction lead to a slightly altered FRET efficiency. While the effect of the photophysical behavior is not very pronounced in this example, it has been demonstrated that these issues, unnoticed, frequently lead to the miscalculation of FRET efficiency [58,59].

### 6.3. Analysis of Weak Protein Interactions Using the Single-Molecule Co-Immunoprecipitation/Pulldown Technique

The co-localization of color-coded interaction partners’ emission signals is the crucial criterion for interaction studies. Recently, this analysis scheme has been extended to include the detection of very weak interactions using the single-molecule pulldown (SiMPull) and real-time single-molecule co-immunoprecipitation method. Even transient interactions, e.g., biomolecular complexes characterized by an affinity up to 1 µM, can be reliably detected using this approach [2,16,129]. Here, native proteins can be directly isolated from cell lysates and immobilized on a glass slide using a biotinylated target-specific antibody. The fundamental advantages of these methods are that (i) biomolecular interactions can be studied that are not amenable to recombinant protein expression and *in vitro* labeling with organic fluorophores; (e.g., many eukaryotic proteins become accessible for the interrogation at the single-molecule level); (ii) the protein is in its fully native state (e.g., all posttranslational modifications are present); (iii) even transient interactions can be observed; (iv) the procedure does not require the purification of the biomolecule but instead can be directly pulled out of a cell lysate which can even be kept in the measurement chamber (e.g., the method saves time and material). In conventional SiMPull experiments, the biomolecular interaction is detected via the co-localization of two different fluorescent signals of the constituting components. In order to detect protein-protein interactions, the molecules are usually detected via the fluorescent signal of a fused fluorescent protein like mCherry or GFP. However, because of the large size of fluorescent proteins (e.g., roughly 27 kDa for eGFP) the positioning of the fluorescent protein has to be carefully chosen. In the further developed variation of this method (termed “real-time coimmunoprecipitation”) weak interaction are detected when the fluorescing interaction partner transiently binds to immobilized but unlabeled interaction partner. In this case, the fluorescing molecule spends more time in the excitation layer than is allowed by pure Brownian motion resulting in a massive burst of photons that can be detected in real-time. This in turn allows the full kinetic characterization of the interaction, e.g., the determination of association and dissociation rates and the lifetime of the complex. Lee *et al.* showed that the shortest lifetime of an interaction has to be 150 ms or longer to be reliably detectable. However, unspecific attachment of the fluorescently-labeled molecule to the surface can occur and has to be carefully controlled to avoid a misinterpretation of data [16]. This method reaches also a limit when long-lived interactions are to be studied as the photoblinking and photobleaching behavior of the fluorescent protein comes into play. Lee *et al.* showed that photoblinking of eGFP occurs every 5 s under typical measurements conditions. Consequently, interactions that exhibit a longer lifetime than 5 s are difficult to study with a 50 ms integration time. However, when decreasing the laser power and increasing the integration time to 400 ms even interactions characterized by a long live-timed could be monitored. Here, the reduced photobleaching of eGFP leads to an extended monitoring time of about 37 s. Taken together, employing the single-molecule co-immunoprecipitation technique interactions with *K*_D_ values ranging from sub-nM to 1 µM can be studied. Given that only minimal amounts of a biomolecule are required for this type of analysis it seems feasible that large cellular interaction networks can be dissected with ease in the future. Moreover, aberrations in protein-interaction patterns typically found in many cancer cells types could be detected.

### 6.4. Single-Molecule FRET Analysis

The fluorescence intensities of donor and acceptor can be calculated from the CCD images and can be recorded over time to retrieve time transients. The FRET efficiency is given as a ratio of the acceptor fluorescence intensity over the sum of the donor and acceptor intensities upon donor excitation [130,131]. Technically, if the FRET efficiency is calculated directly from fluorescence intensities retrieved from single-molecule spectroscopy without γ-corrections, the resulting value is termed the proximity ratio (PR):

(4) $$PR=\frac{I_{D_{Ex}}^{A^{Em}}}{I_{D_{Ex}}^{A^{Em}}+I_{D_{Ex}}^{D^{Em}}}$$

The fluorescence intensities of the donor and the acceptor upon donor excitation are expressed in $I_{D_{Ex}}^{A^{Em}}$ and $I_{D_{Ex}}^{D^{Em}}$. In order to obtain the real FRET intensity, the PR has to be corrected for crosstalk (LK, leakage of the donor emission into the acceptor channel) and for direct excitation (DIR, direct excitation of the acceptor upon donor excitation) as well as for differences in brightness and the detection efficiency of the setup for the two dyes which is expressed in the so called γ-factor [88].

(5) $$\begin{array}{l} {I^{*}}_{D_{Ex}}^{A^{Em}}=I_{D_{Ex}}^{A^{Em}}-LK-DIR\;\text{and}\;\gamma =\frac{\Phi_{A}\cdot \eta_{A_{Ex}}^{A^{Em}}}{\Phi_{D}\cdot \eta_{D_{Ex}}^{D^{Em}}}\\ E=\frac{{I^{*}}_{D_{Ex}}^{A^{Em}}}{\gamma \cdot I_{D_{Ex}}^{D^{Em}}+{I^{*}}_{D_{Ex}}^{A^{Em}}} \end{array}$$

**Figure 7:**
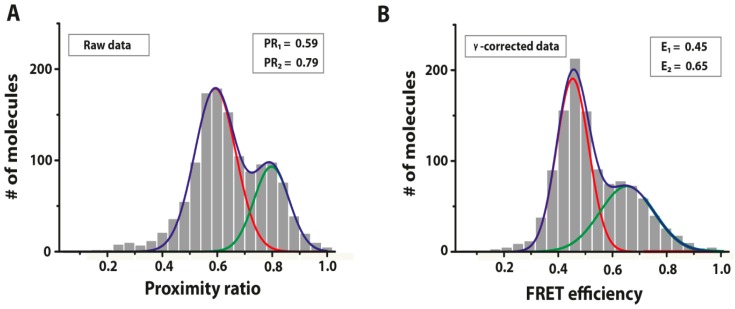
Analysis of single-molecule data to retrieve quantitative distance information. (**A**) After correction of the fluorescence intensities values for direct excitation of the acceptor with the donor excitation laser and cross-talk of the donor emission into the acceptor detection channel a proximity ratio histogram can be obtained. The exemplary histogram was obtained from a protein with two different conformations (unpublished data) resulting in two populations. The data are fitted with two Gaussian s with peaks at 0.59 and 0.79; (**B**) In order to retrieve absolute FRET values a γ -correction has to be carried out. Gaussian fitting of the resulting γ-corrected FRET efficiency histogram reveals that the corrected FRET efficiencies drastically deviate from the uncorrected data (E = 0.45/0.69).

Φ denotes the quantum yields of the two dyes and η are their detection efficiencies with the microscope used for the measurement. The detection efficiencies and the quantum yields are combined to the correction factor γ. The corrected FRET efficiencies can be used to retrieve real distances (Equation (2)). Figure 7A,B exemplifies the crucial difference between PR and FRET efficiency once the γ-correction has been carried out. However, changes in distances can be equally well followed using the PR when the gamma value remains unaltered during the measurement.

Alternatively, the FRET efficiency can be calculated from the donor fluorescence lifetime in the presence (*τ_D_*_(_*_A_*_)_) and absence (*τ_D_*_(0)_) of the acceptor [132]:

(6) $$E=1-\frac{\tau_{D(A)}}{\tau_{D(0)}}$$

With the calculated values a FRET efficiency histogram can be created where molecules are classified according to their FRET efficiency. A histogram provides four features that provide further information: the number of peaks, the width of the population, the mean peak position and area under the peaks. Commonly single-pair FRET (spFRET) is employed to detect inter- or intramolecular conformational changes. Different conformations usually exhibit different mean FRET efficiencies (mean peak position of the population) if there is a dynamic equilibrium between the states. Figure 4C shows the dynamics of a DNA Holliday junction, which is easily detectable in form of the fluctuating FRET values (blue trace). The example given in Figure 4 shows two main FRET efficiencies of 0.29 and 0.7. The higher value is assigned to the Iso_II_ conformation and the lower one to the Iso_I_ confirmation.

The widths of the FRET efficiency distribution in a population is by nature broadened by shot noise, rotational freedom of the bound dyes (as κ^2^ is changing) and by conformational fluctuations of the labeled molecule. The primary source of broadening is shot noise, the Poisson (shot) noise fluctuations of a single molecule [133]. This effect is especially pronounced at low photon count numbers and an intrinsic feature of a measurement with limited amount of data available per molecule. Considering the burst size distribution, one can check if there is additional broadening beyond shot noise [89]. Restrictions in the rotational freedom of the bound dyes can also be a reason for broadening of the of the distribution width. The rotational freedom of the dye is essential so that the dye can sample all relative dipole orientations much faster than the timescale of the measurement (integration time) and κ^2^ averages over time to 2/3. As discussed before, the rotational freedom is not always ensured even though dyes are typically linked via saturated carbon-carbon linkers of a length of around 10 Å to the molecule of interest.

If the rotation of the dye is restricted and the fluctuation between different states approaches the timescale of the measurement, multiple FRET populations will be measured that do not originate in different conformational states of the biomolecule. However, one of the main reasons for broadening are conformational fluctuations in the labeled molecule. Especially larger macromolecules are often not defined by a single rigid structure, but rather by an ensemble of conformational similar states. The width of the peaks in the histogram informs about the timescale of the conformational rearrangements of the molecules. If the rearrangement of the molecules is slower than the timescale of the measurement, the width of the peaks is strongly influenced and in the extreme this would lead to the generation of multiple peaks. If the rearrangement is much faster than the measurement timescale the effect is averaged out. More sophisticated analysis tools like photon distribution analysis (PDA) of the spFRET data can reveal whether broadening of the histograms can be attributed to structural heterogeneity or dye artifacts [134,135,136]. The ratio of the peak areas reflects the equilibrium population of the two states. With this type of analysis it is possible to link single-molecule to ensemble data as shown in a number of studies [137,138].

A major concern when measuring dynamic processes like conformational changes with diffusion based spFRET is the time resolution. Slow conformational changes would mean that each molecule is either folded or unfolded when diffusing through the confocal volume. This would lead to clearly resolved peaks in the histogram interpreted as two individual species with static heterogeneity. The peak widths provide some information about the dynamics within the state. However, if the conformational change between the two states is so rapid that both states are sampled multiple times when diffusing through the observation volume, the dynamic heterogeneity in the solution is not detectable. The faster the process, the greater the averaging of the FRET efficiencies till the two states cannot be resolved with ALEX any more. However, fluorescence correlation spectroscopy (FCS) extends to the low nanosecond range (ns-FCS) [139] and has been used to monitor for example ultra-fast protein folding. As the combination of FRET and FCS is very demanding, it is not part of this introductory article. Here we would like to refer to a review written by Sahoo and Schwille [98].

Often a third peak is shown in a spFRET histogram called the “zero peak”. This peak is generally attributed to molecules lacking an acceptor dye or is accounted to acceptor dyes that prematurely undergo a bleaching event. Low FRET efficiency populations can easily be hidden underneath the “zero peak”. The ALEX scheme represents a solution for this problem [90]. In addition to the FRET efficiency, the presence and status of the acceptor dye is probed in parallel by directly exciting the acceptor. This excitation scheme allows the calculation of the stoichiometry or brightness ratio S:

(7) $$S=1-\frac{I_{D}^{D}+I_{D}^{A}}{I_{D}^{D}+I_{D}^{A}+I_{A}^{A}}$$

**Figure 8:**
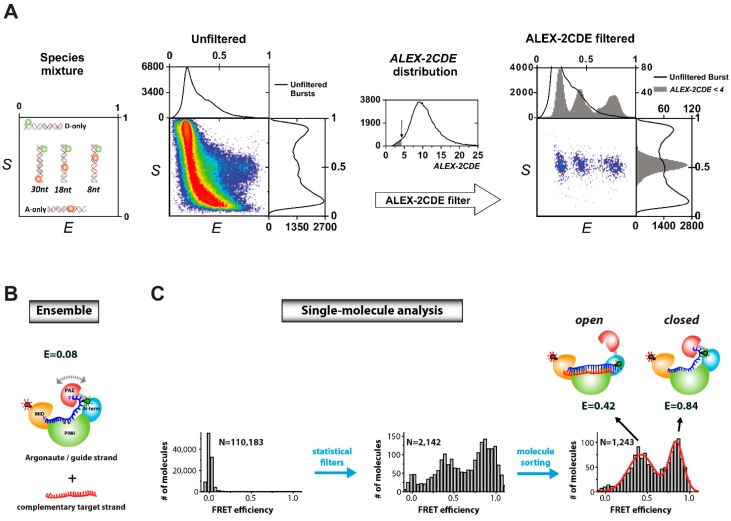
(**A**) Advanced statistical analysis of single-molecule FRET data acquired using confocal microscopy with alternating laser excitation (ALEX) [142]. The ALEX-2CDE filter uncovers a minor, doubly labeled dsDNA subpopulation hidden in the larger concentrations of singly labeled dsDNA subpopulations [30 pM donor-labeled and 30 pM acceptor-labeled ssDNA with three different doubly labeled dsDNA, 6 pM each (E = 0.22, E = 0.46, and E = 0.85)]. (Adapted from Tomov, T. E. *et al.*, 2012 “Disentangling subpopulations in single-molecule FRET and ALEX experiments with photon distribution analysis”. Biophysical Journal 102(5): 1163–1173); (**B**) In order to understand the conformational transitions of the Argonaute-protein a donor-acceptor pair (AttoATTO550/Alexa647) was engineered into the protein-nucleic acid complex. In ensemble measurements a surprisingly low FRET efficiency of 0.08 was determined; (**C**) When measured at the single-molecule level a comparable low FRET efficiency was measured. However, unlike in ensemble, the single-molecule approach revealed that the low FRET efficiency is due to a large access of donor-only and acceptor-only labeled molecules in the sample. Statistical filters gave access to the 1% of molecules that exhibit a stable FRET signal. Ultimately, this revealed that Argonaute adopts a different conformation (E = 0.42) when the target strand is loaded as compared to the Argonaute-guide DNA only complex, which is characterized by a high FRET value (E = 0.84).

Donor-only labeled molecules will exhibit an S value of 1, acceptor-only labeled molecules an S value of 0 and donor-acceptor-labeled molecules will exhibit an S value of ideally 0.5 (depending on the relative excitation intensities). It is advised to choose FRET pairs with similar brightness (*i.e*., amount of photos emitted while diffusing through the confocal volume) in order to assure S = 0.5 at laser excitations within a similar power range (100–200 µW). E/S values are commonly plotted in a two dimensional histogram (Figure 8A) where the different populations can be easily distinguished. Common pairs for confocal single-molecule FRET are Alexa488-Alexa647, ATTO550-ATTO647N or Cy3-Cy5. Ultimately, this scheme allows the sorting of single molecules according to their fluorescence parameters E and S. Only molecules with an S value of 0.5 (indicating the presence of donor and acceptor) can now be considered for FRET efficiency analysis. Alternatively, a weak but continuous direct excitation of the acceptor fluorophore can be implemented. This adds a constant fluorescence to the FRET signal which allows the experimenter to distinguish between no FRET (only the signal from the direct excitation) and no acceptor fluorescence present (no signal in the FRET channel) [140]. This approach offers twice the time resolution as compared to ALEX but lessens the SNR of the FRET channel and requires a high sample homogeneity and a constant acceptor fluorescence over the course of the measurement. Nevertheless, with both techniques molecules without an acceptor dye can be excluded from the analysis which leads to the removal of the zero peak and low FRET values are measurable without interference. Absolute FRET efficiencies can be calculated if corrections are carried out taking into account the background contribution, direct excitation and leakage contribution and the correction factor γ (for details of correction of confocal data the reader is referred to [88,91] and for correction of TIRF data to [141]). Moreover, ALEX can also be used to gain information about the dye stoichiometry for affinity or integrity measurements. Recently, additional selection criteria have been developed that enable a further filtering of FRET and FRET/ALEX data based on photon distribution analysis [142]. This analysis takes the FRET and brightness ratio distributions in a burst into account. Filtering of the data according to the brightness ratio (ALEX-2CDE filter) is used to identify and remove blinking, bleaching and donor/acceptor-only events. A combination of both filters helps to retrieve more reliable and accurate FRET data and even allows measurements at higher concentrations than commonly used since the filters remove partially coinciding bursts.

The outstanding potential of this filtering process, which allows the identification of molecules even if they only represent as a small fraction of all molecules in the sample has been recently exploited to describe the dynamic behavior of the Argonaute protein. Here, it was possible to investigate the conformation of the protein-nucleic acid complex from a heterogeneous mixture of molecules with a large excess of donor- and acceptor-only labeled species present. When measured at the ensemble level, the unfiltered dataset resulted in a mean FRET efficiency of 0.08 due to the over-representation of donor-only labeled molecules. Even when measuring a large set of molecules at the single-molecule level, a very low apparent FRET efficiency value was observed at first. However, after removal of all impurities and partially labeled molecules as well as blinking and bleaching effects, only 1% of the originally detected molecules were used for the FRET efficiency analysis [143], which revealed two different conformations of the complex centered at a mean FRET efficiency of 0.42 and 0.84 (Figure 8B,C). Another example is shown in Figure 8A. Here, the individual population in a complex mixture of donor-only and acceptor-only DNAs (in 5 fold excess over the doubly-labeled DNA) and three different doubly-labeled DNAs could be efficiently disentangled when using the ALEX-2CDE filter but remained obscured when leaving the dataset unfiltered.

## 7. Perspective

Currently, enhanced efforts are made to develop minimally invasive labelling schemes, to further increase the photostability of fluorescent dyes and to expand the range of concentrations suitable for single-molecule measurements. Labeling via unnatural amino acids incorporated into the protein of interest has become a feasible and efficient strategy to site-specifically introduce fluorescent probes. Advanced coupling strategies include for example the “strain-promoted click reaction” and Staudinger Ligation [10,144,145,146]. On the fluorophore side “self-healing” dyes have been introduced [147,148]. Here, the photostabilizing reagent is covalently attached to the dye leading to an enhanced photostability. From some near infrared fluorescent dyes such as ATTO647N several million photons can be collected [66]. In contrast, photostabilization is not as efficient for dyes excited at shorter wavelengths (e.g., Alexa488) preventing efficient photon collection. Further developments in this area will bring about a better control of dye photophysics, but another issue is becoming more and more restricting. One of the reasons why optical single-molecule techniques have not found widespread distribution in the laboratory yet is that the concentration limit prevents the investigation of the vast majority of biological interactions. Practically speaking, this barrier can be overcome by for example reducing the effective observation volume e.g., using lipid vesicles. Alternatively, nanophotonic structures have been utilized to either further confine the observation volume (e.g., zero-mode waveguides) or to enhance the fluorescence of a single-molecule attached in a measurement hot-spot (reviewed for example here [149,150]). Zero-mode waveguides are currently utilized for single-molecule sequencing instruments [151]. Recently, this instrument has been extended to be compatible with virtually any biological sample enabling the automated high-throughput multiplexed monitoring of dynamic biological processes [152]. Moreover, it already integrates solutions for most of the challenges encountered in single-molecule microscopy experiments. e.g., only minimal sample volume of 25–30 µL required, a nitrogen-atmosphere ensures oxygen-free measurement conditions preventing fast dye bleaching which results in extended measurement duration up to several minutes. Although this approach solves many technical issues, the dye coupling chemistries restrict single-molecule measurements often to robust proteins and macromolecular complexes that can be easily produced in a recombinant form prior to labelling. Sensitive biological interactions are incompatible with single-molecule interrogation so far. Recent efforts aim to access the physiological status of biological systems and to reach out to explore the complexity of cellular systems. In one strategy fluorescently labeled molecules were electroporated into bacterial cells. Both labelled DNA and DNA-binding proteins still acted as a reliable fluorescent reporter once when re-introduced into the bacteria [153,154]. Minimally invasive approaches include the “single-molecule pulldown” technique. While this method relies on immobilized molecules, a new single-molecule fluorescence approach allowed the direct observation of interactions and stoichiometries in caveolae complexes from cell extracts in solution [95]. Gambin *et al.*, analyzed the self-assembly and caveolar association of mCherry- and GFP-tagged cavin proteins in solution employing. These studies demonstrate that fluorescence-based single-molecule approaches now enter a new era where the physiological complexity of biological systems can be studied.
